# Detailed Investigation of Factors Affecting the Synthesis of SiO_2_@Au for the Enhancement of Raman Spectroscopy

**DOI:** 10.3390/nano12173080

**Published:** 2022-09-05

**Authors:** Nguyen Thi Phuong Thao, Loc Ton-That, Cong-Thuan Dang, Jan Nedoma

**Affiliations:** 1 Department of Telecommunications, VSB Technical University of Ostrava, 708 00 Ostrava, Czech Republic; 2 Future Materials & Devices Laboratory, Institute of Fundamental and Applied Sciences, Duy Tan University, Ho Chi Minh City 700000, Vietnam; 3 Faculty of Natural Sciences, Duy Tan University, Da Nang City 550000, Vietnam

**Keywords:** SiO_2_@Au, core shell, Raman spectroscopy, SERS

## Abstract

The reaction time, temperature, ratio of precursors, and concentration of sodium citrate are known as the main factors that affect the direct synthesis process of SiO_2_@Au based on the chemical reaction of HAuCl4 and sodium citrate. Hence, we investigated, in detail, and observed that these factors played a crucial role in determining the shape and size of synthesized nanoparticles. The significant enhancement of the SERS signal corresponding to the fabrication conditions is an existing challenge. Our study results show that the optimal reaction conditions for the fabrication of SiO_2_@Au are a 1:21 ratio of HAuCl_4_ to sodium citrate, with an initial concentration of sodium citrate of 4.2 mM, and a reaction time lasting longer than 6 h at a temperature of 80 °C. Under optimal conditions, our synthesis process result is SiO_2_@Au nanoparticles with a diameter of approximately 350 nm. In particular, the considerable enhancement of Raman intensities of SiO_2_@Au compared to SiO_2_ particles was examined.

## 1. Introduction

Recently, the surface plasmon resonance properties of metal nanostructures have attracted significant attention from researchers because of their potential applications in various fields, e.g., materials science [1], biology [2], chemistry [3], and environmental analysis [4]. The plasmonic properties of metal nanoparticles significantly depend on the shape, size, and nature of the nanostructured materials employed [5,6]. Among applications based on the surface plasmon phenomena, surface-enhanced Raman scattering (SERS) has emerged as a strong analytical technique that considerably contributes to the enormous improvement of SERS-based sensing techniques [7,8]. SERS is a novel optical technique that combines nanotechnology with Raman scattering spectroscopy to produce an analytical tool with high sensitivity. It is based on the excitation of surface plasmon resonance to amplify the Raman signal on the metal surface [9]. Modern SERS techniques have been widely developed in recent years and opened a new step in many scientific fields, such as the environment, food, and biomedicine [10,11]. The SERS sensing technique has become a powerful analytical tool for researchers in the nanotechnology field to identify the component composition of substances and provide information about their molecular structure at low concentrations without destroying them [12,13,14]. In this technique, the SERS substrate plays a crucial role in determining the correction and effectiveness of the analytical tool. Therefore, developing new materials and optimal nanostructures is necessary to produce stable and efficient substrates for SERS. The nanostructures of noble metals, such as Au [15,16], Ag [17,18], and Cu [19,20], have been studied as excellent plasmonic materials and widely employed in plasmonic applications. Such nobble metals exhibit surface plasmonic resonances in the visible and NIR region [21]. 

The core-shell nanostructures have been discovered as efficient SERS substrates to amplify the Raman signal in which the cores are SiO_2_ spherical particles, and their shell components are composed of noble metal nanoparticles. The position of the surface plasmon band of core-shell nanostructures can be tuned according to the size, shape, and surface morphology [22,23]. Among these substrates, SiO_2_@Au nanoparticles have been extensively studied in many chemical and biomedical research fields over the past year due to their unique optoelectronic and physicochemical properties [24,25].

However, synthesizing a complete metal shell with uniform and high coverage around the silica core is challenging. Therefore, a series of strategies for the synthesis of SiO_2_@Au nanoparticles on SiO_2_ have been developed, such as gold electroless plating [26], the self-assembled monolayer method [27], the layer-by-layer cross-linking method [28], controlled seed growth [29], the ultrasound-assisted Stober method [30], and the sol-gel method [31]. The most common method for synthesizing SiO_2_@Au core-shell structure nanoparticles is through a two-step process that involves decorating a functionalized SiO_2_ surface with small gold seeds and the growth of a gold shell. Additionally, some studies have produced SiO_2_@Au nanoparticles through the direct growth of gold shells on SiO_2_ spheres without gold seeds. Michael et al. proposed coating a thin and uniform gold layer on bare silica nanoparticles by reducing a gold (I) chloride solution dissolved in acetonitrile with ascorbic acid [32]. Zhang et al. reported a facile method with a one-pot, one-step process for preparation SiO_2_@Au by heating a solution containing chloroauric acid (HAuCl_4_), 2-methylaminoethanol (2-MAE), CTAB, and TEOS at 80° [33].

In some approaches related to the deposition of gold seeds on SiO_2_ spheres, the functionalization of nanoparticles is essential to increase the absorption of Au seeds on the SiO_2_ surface and maintain the stability of the nanostructure during the synthesis process. The coverage level of the gold nanoparticles on the silica core is affected by the strength of attraction between the gold particles and the core, as well as by the force balance between the particles in the shell region [34,35]. Therefore, the surface of SiO_2_ spheres is commonly functionalized by organic agents with high adhesion with gold, such as (3-aminopropyl) trimethoxysilane (APTMS), polyethyleneimine (PEI), and 3-mercaptopropyltrimethoxysilane (MPTMS) [36,37]. 

An approach involving an amine-terminated coupling agent, which acts as an adhesive agent to attach gold nucleation to silica nanoparticles, was first proposed by Halas et al. [38]. The controlled shell growth method involving small gold seeds (diameters of 1 to 2 nm) acting as nucleation center on the surface of SiO_2_ spheres was proposed by Yukhymchuk et al. In this study, SiO_2_ spheres with a diameter of 160 to 210 nm were functionalized with positively charged amino groups before decoration with gold nanoparticles. A complete gold shell was formed by reducing gold salt with reducing agents of hydroxyl-amine muriatic NH_2_OH.HCl and sodium borohydride NaBH_4_ [39]. Moreover, Kandpal et al. synthesized SiO_2_@Au nanoparticles with a multistep process involving the combination of the citrate and borohydride methods. The authors started with the citrate method, reducing the HAuCl_4_ gold salt with trisodium citrate Na_3_(C_6_H_5_O_7_) at 80 for 30 min for deposition of gold nucleation on functionalized silica spheres with 3-aminopropyltriethoxysilane (APS). Then, the borohydride method was applied to develop gold nucleation for formation a gold shell, which reduces HAuCl_4_/K_2_CO_3_ solution with sodium borohydride (NaBH_4_). The obtained results demonstrate that the combination of the two methods resulted in the significant enhancement of the SERS signal compared to each of the methods alone [40]. The technique of grafting Au nanoparticles on trimethoxysilane (APTMS)-functionalized SiO_2_ spheres and then priming these nanoparticles with gold colloids of 2 nm and 10 nm before chemically reducing the gold salt with NaBH_4_ (5.3 mM) was investigated by Saini et al. The thickness of the gold shell is controlled by the ratio between the precursor particles and the gold salt solution. This method was proven to improv the SERS signal with a small amount of gold [41]. Wang et al. reported a fabrication process of SiO_2_@Au nanoparticles using the isotropic growth method of gold seeds (3–5 nm) on PEI-functionalized SiO_2_ spheres (diameter of 190 nm) by reducing gold salt with hydroxylamine hydrochloride (NH2OH.HCl) under ultrasound assistance instead of traditional methods. This approach successfully reduced the fabrication time and the time for completing the gold shell within 5 min. Moreover, the attained SiO_2_@Au nanoparticles were highly homogeneous and well-defined in size and shape [42]. Khurana et al. proposed synthesizing freckled SiO_2_@Au nanocomposites (NCs) by gold-seed-mediated growth on MPTMS-functionalized silica spheres. Freckled SiO_2_@Au NCs with SiO_2_ core sizes of 880 nm and 430 nm and a shell thickness in the range of 12–50 nm exhibited high surface consistency of the shell and excellent enhancement of the SERS signal [14]. Table 1 presents a review of the methods and related conditions for the fabrication of SiO_2_@Au. Although reports have presented the preparation process of Au@SiO_2_ using different methods, as well as the strong points of each method, no reports have been published showing factors affecting the synthesis of SiO_2_@Au nanomaterial in detail.

In this study, we present a deep view of factors that affect the synthesis of SiO_2_@Au material, including temperature, reaction time, the ratio of precursors, and the concentration of sodium citrate. The characteristics of the obtained SiO_2_@Au nanoparticles will be investigated using UV-Vis spectra, SEM, and DLS. The Raman spectroscopy of SiO_2_ spheres before and after coating Au nanoparticles will also be examined.

## 2. Materials and Methods

### 2.1. Materials

Non-functionalized Silica microspheres approximately 0.27 µm in the dry form with 100% solids were purchased from (Polyscience Asia Pacific Inc., Taipei, Taiwan). The surface group of non-functionalized SiO_2_ is normally silanol (SiOH), and the refractive index is around 1.43–1.46 (589 nm).

Gold (III) chloride solution (HAuCl_4_, 99.9%), polyethylenimine (-[-CH_2_-CH_2_-NH-]-), and sodium citrate dihydrate (HOC(COONa)(CH_2_COONa)_2_ 2H_2_O) were purchased from of Sigma-Aldrich, St. Louis, MO, USA commercial suppliers. The gold (III) chloride solution has a composition Au of 17 wt.% and concentration of 30 wt.% in dilute HCl. Sodium citrate dihydrate is the trisodium salt of citric acid, which takes in citrate (3−). All chemicals were of analytical grade and used without further purification. 

### 2.2. Preparation of SiO_2_@Au

**Scheme 1:**
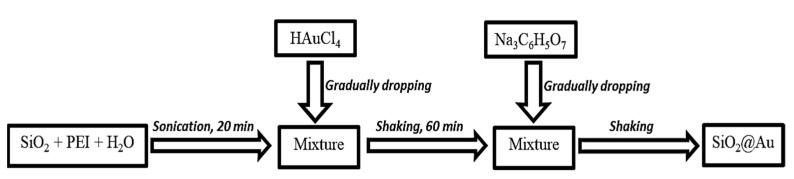
Process of preparing SiO_2_@Au material.

SiO_2_@Au material was synthesized via chemical reduction pursuant to Scheme 1. A mixture of 0.01-g SiO_2_ spheres (approximately 270 nm in size) and PEI was dispersed in DI water under sonication for 20 min. Next, an HAuCl_4_ solution was gradually dropped into the mixture. The obtained mixture was then shaken for 60 min before adding sodium citrate at the investigated temperature. Factors affecting the preparation were examined, such as temperature, reaction time, the ratio of precursors, and the concentration of sodium citrate.

### 2.3. Characterization

The extinction spectra of the synthesized SiO_2_@Au nanoparticles were determined using a UV-Vis spectrometer V630 (V630, Jasco, Japan). This instrument operates with high speeds of up to 8000 nm/min and can measure wavelength ranges from 190 to 1100 nm. The images of surface morphologies of SiO_2_, SiO_2_@PEI, and SiO_2_@Au microspheres were observed and evaluated by a scanning electron microscope (S-4800 SEM, Hitachi, Japan) with an accelerating voltage range the electron beam in the range of 0.5 to 30kV. A DLS analytical instrument (Horiba, SZ-100Z2, Kyoto, Japan) was used to determine the size of SiO_2_ and SiO_2_@Au by measuring the intensity of the dynamic light scattering of these particles. The Raman scattering intensity was measured on a Raman spectrometer (Horiba Ihr 550, Kyoto, Japan) using a laser source with an excitation light wavelength of 532 nm and methylene blue analyte.

## 3. Results

### 3.1. Factors Affecting the Synthesis of SiO_2_@Au

#### 3.1.1. Effect of Temperature

**Figure 1:**
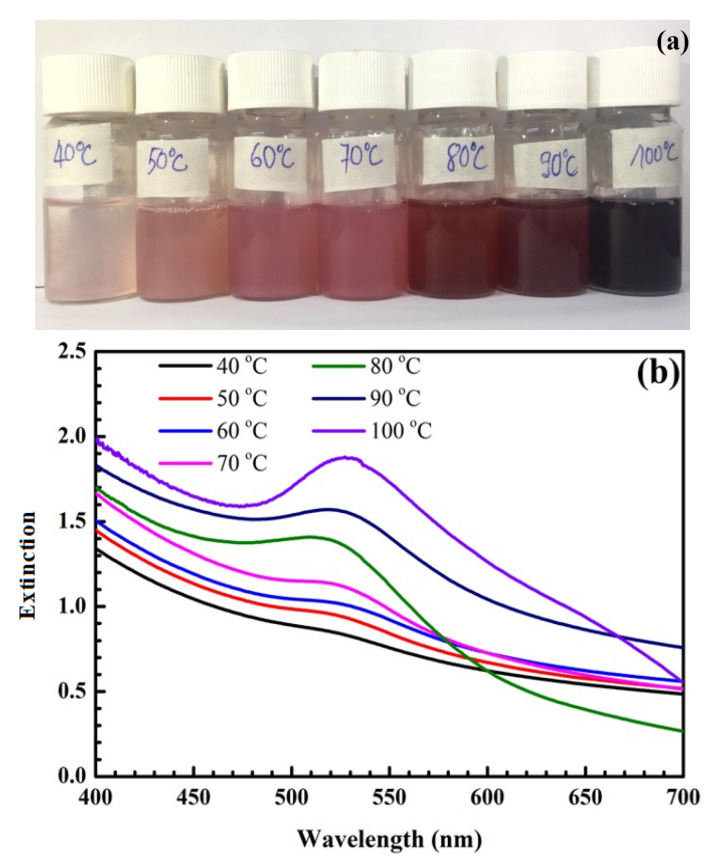

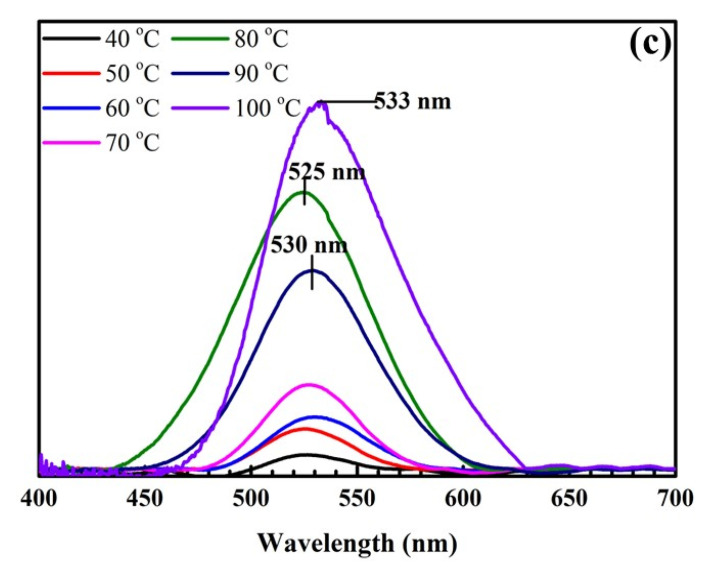
Pictures of samples at different temperatures (**a**). UV-Vis spectra of samples at different temperatures (**b**) and their UV-Vis baseline spectra (**c**).

Figure 1 shows pictures of samples at different temperatures and their UV-Vis spectra. As shown in Figure 1a, gold nanoparticles (AuNPs) are formed with a characteristic color range from light pink to dark purple, corresponding to the surface plasmon resonance (SPR) peaks between 520 and 535 nm shown in Figure 1b,c. When the temperature increases from 40 °C to 80 °C, the sample color changes from pale pink to red, and the maximum extinction wavelength (λ_max_) increases from 520 to 525 nm (Figure 1b). In this study, our goal was to synthesize AuNPs 40 nm in size related to the wavelength of 525 nm, so the chosen optimal temperature was 80 °C.

#### 3.1.2. Effect of Reaction Time

**Figure 2:**
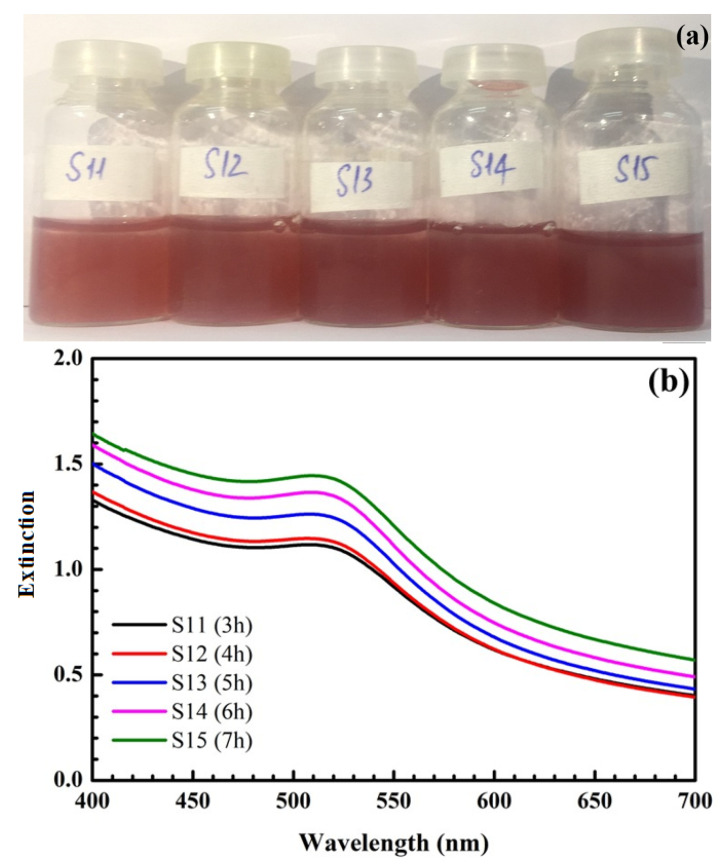

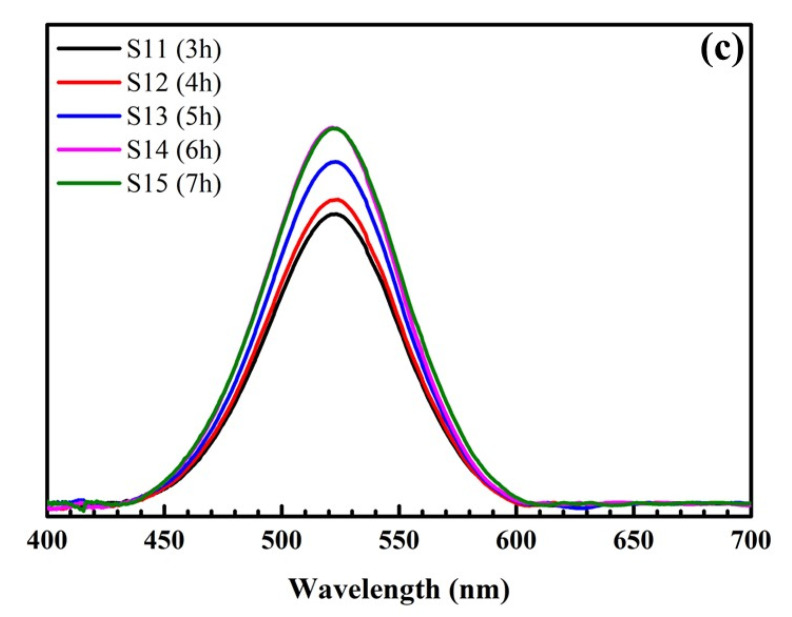
Pictures of samples at different reaction times (**a**). UV-Vis spectra of samples at different reaction times (**b**) and their UV-Vis baseline spectra (**c**).

Figure 2 shows the formation of SiO_2_@Au at different reaction times from 3 to 7 h. Clearly, the color of the synthesized solution changes from light red to dark red, and the maximum extinction wavelength is within the range of 524 nm to 525 nm. Additionally, the intensity of peaks increases from 3 to 6 h and remains stable after 6 h.

#### 3.1.3. Effect of the Ratio of HAuCl_4_ to Sodium Citrate

**Figure 3:**
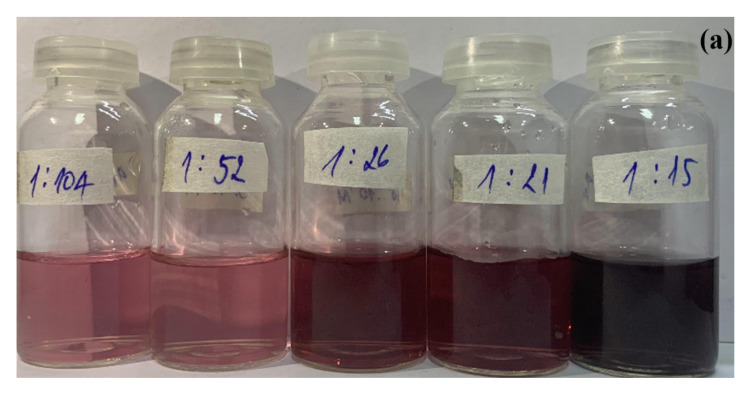

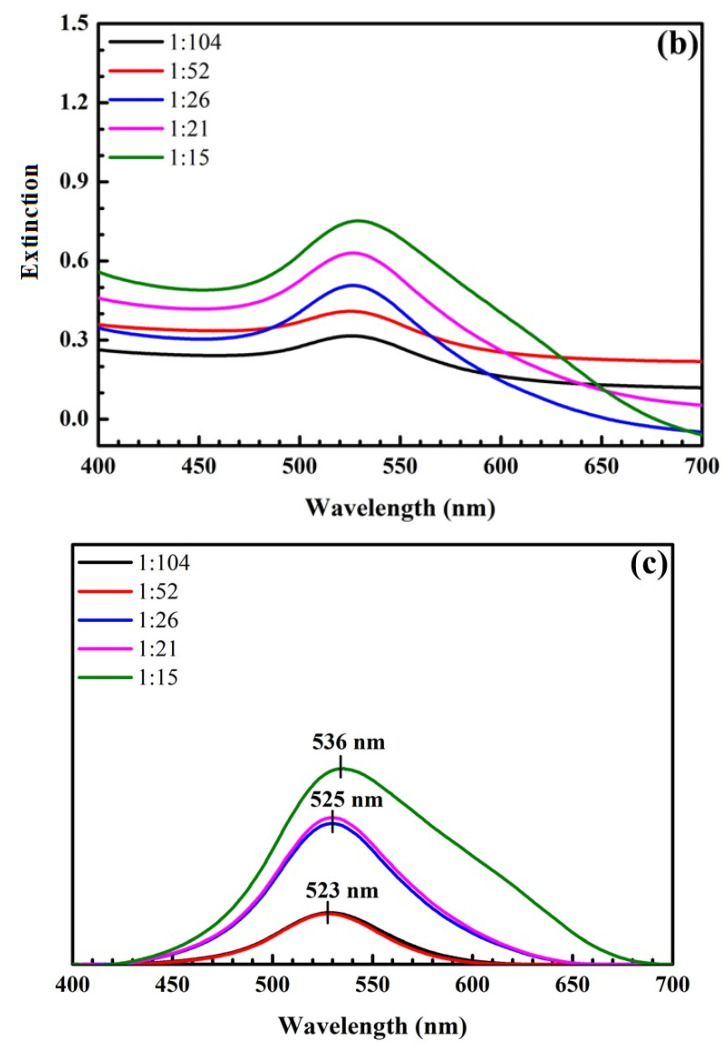
Pictures of samples at different ratios of HAuCl_4_ to sodium citrate (**a**). UV-Vis spectra of samples at different ratios of HAuCl_4_ to sodium citrate (**b**) and their UV-Vis baseline spectra (**c**).

The pictures and UV-Vis spectra of SiO_2_@Au shown in Figure 3 demonstrate that the color of SiO_2_@Au solution changes from pink to burgundy when the ratio of HAuCl_4_ to sodium citrate changes 1:104 to 1:15, corresponding to the maximum extinction wavelength within the range of 523–536 nm.

#### 3.1.4. Effect of the Concentration of Sodium Citrate

**Figure 4:**
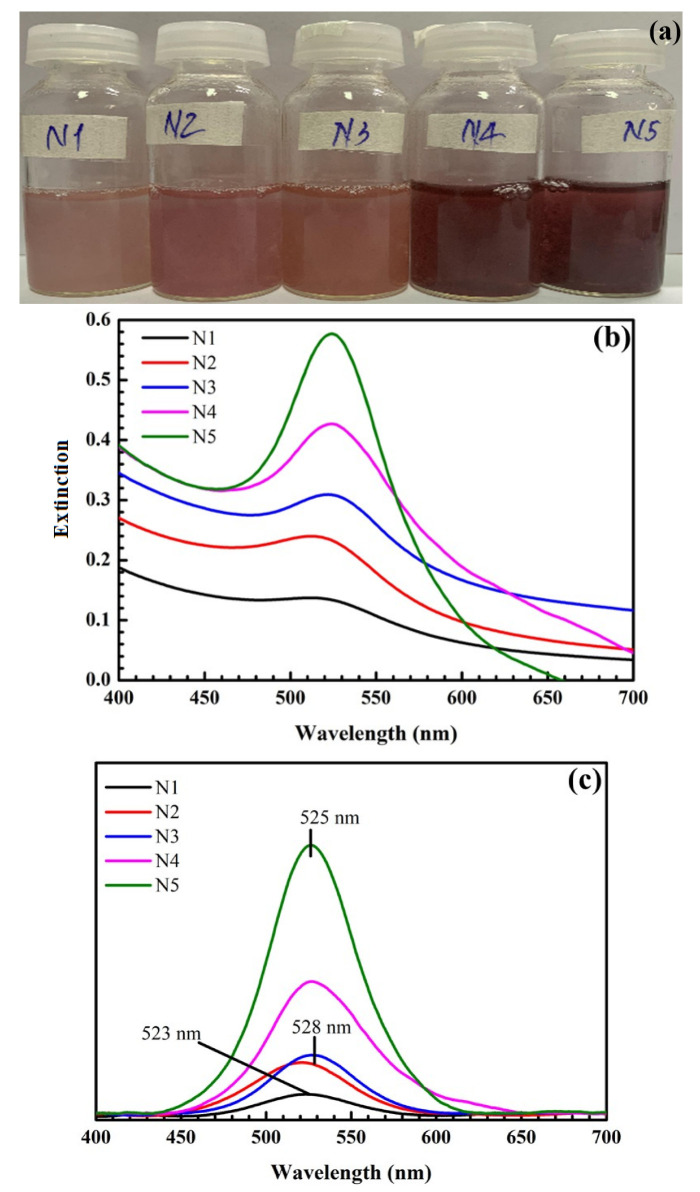
Pictures of samples at different concentrations of sodium citrate (**a**). UV-Vis spectra of samples at different concentrations of sodium citrate (**b**) and their UV-Vis baseline spectra (**c**). N1: 0.21 mM; N2: 0.42 mM; N3: 0.84 mM; N4: 1.5 mM; N5: 4.2 mM.

Sodium citrate was chosen as a reducing agent, so it directly affects the reaction rate and amount of synthesized AuNPs. As shown in Figure 4, when the concentration of sodium citrate is increased, the color of the obtained solution changes from light pink to dark red, and the intensity of the SPR peaks increases.

### 3.2. Characterizations of Obtained SiO_2_@Au

Based on the obtained results reported above, the optimal conditions for the synthesis of SiO_2_@Au are a ratio of HAuCl_4_ to sodium citrate of 1:21, an initial concentration of sodium citrate of 4.2 mM, and a temperature of 80 °C for 6 h. Characteristics of SiO_2_@Au are determined using UV-Vis spectra, SEM images, and DLS.

**Figure 5:**
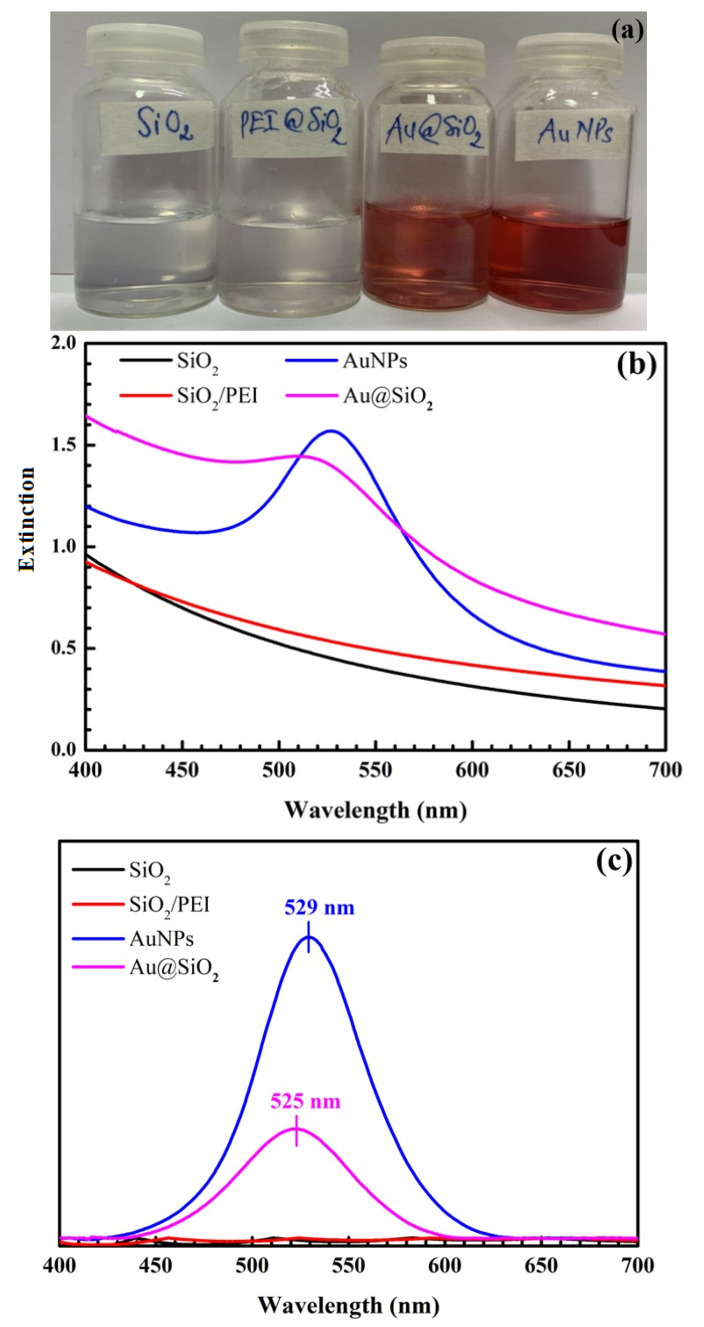
Pictures of SiO_2_, SiO_2_@PEI, AuNPs, and SiO_2_@Au synthesized under optimal conditions (**a**). UV-Vis spectra of SiO_2_, SiO_2_@PEI, AuNPs, and SiO_2_@Au synthesized under optimal conditions (**b**) and their UV-Vis baseline spectra (**c**).

Figure 5 presents pictures of SiO_2_, SiO_2_@PEI, AuNPs, and SiO_2_@Au synthesized under optimal conditions and their UV-Vis spectra. Notably, there is no SPR peak of SiO_2_ and SiO_2_@PEI, whereas the maximum extinction wavelengths of AuNPs and SiO_2_@Au are at 529 nm and 525 nm, respectively.

**Figure 6:**
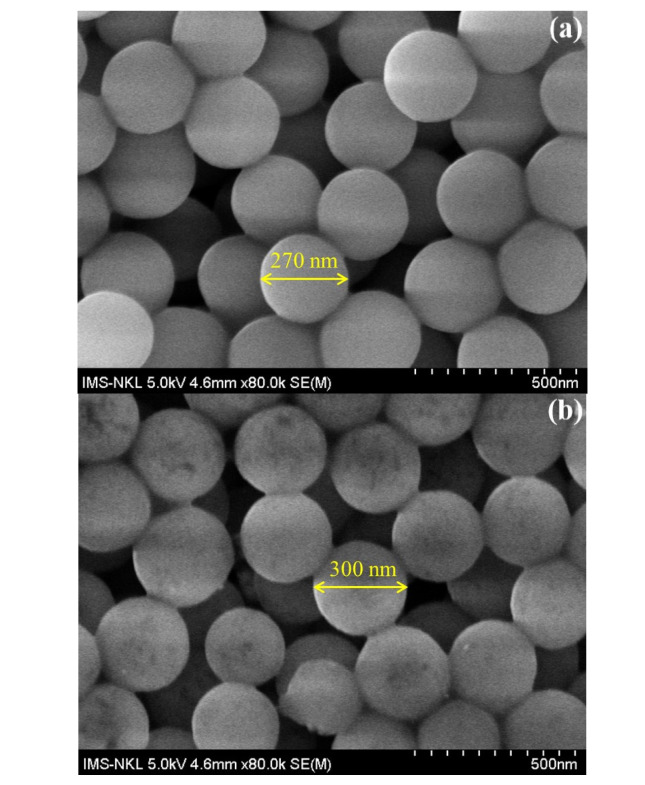

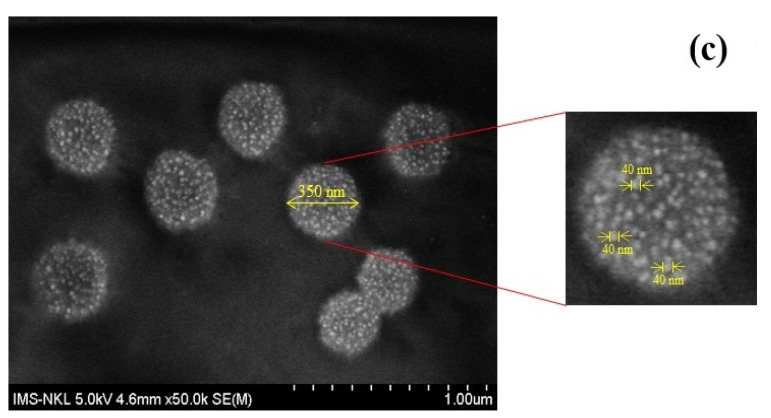
SEM images of SiO_2_ (**a**), SiO_2_@PEI (**b**), and SiO_2_@Au (**c**).

The morphologies of SiO_2_, SiO_2_@PEI, and SiO_2_@Au are shown in Figure 6. The average size of SiO_2_, SiO_2_@PEI, and SiO_2_@Au particles is 270 nm, 300 nm, and 350 nm, respectively.

**Figure 7:**
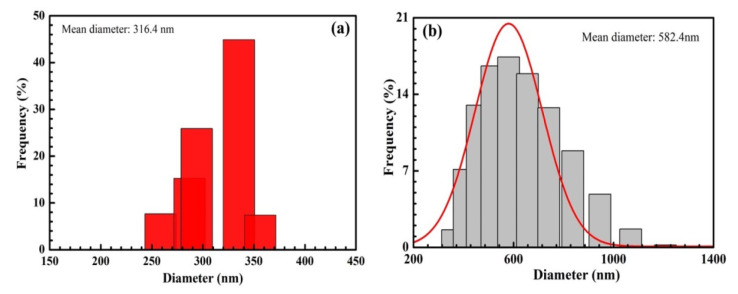
Dynamic Light Scattering results of SiO_2_ (**a**) and SiO_2_@Au (**b**).

According to the DLS results of SiO_2_ and SiO_2_@Au shown in Figure 7, the size of SiO_2_ particles ranges from 260 to 360 nm, and the average size of SiO_2_ particles is about 316 nm. Similarly, the average size of SiO_2_@Au particles is approximately 582.4 nm.

### 3.3. Enhancement of Raman Spectroscopy

**Figure 8:**
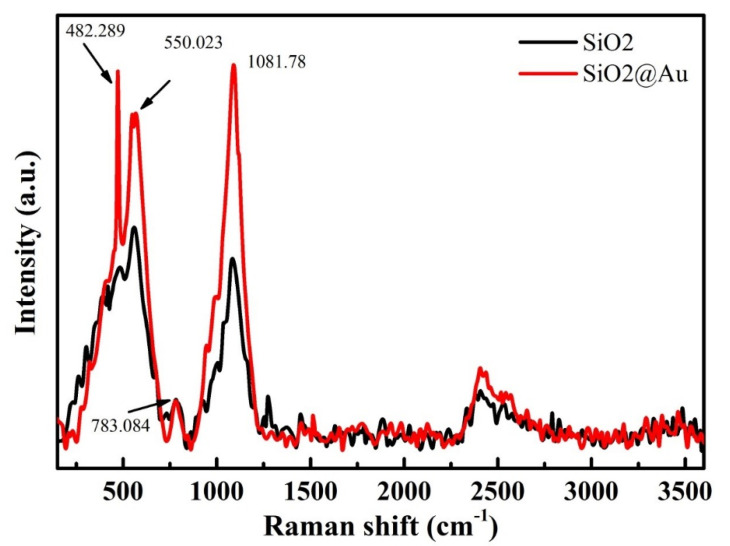
Raman spectra of methylene blue solution deposited on SiO_2_ and SiO_2_@Au.

Figure 8 shows Raman spectra of methylene blue molecules deposited onto SiO_2_ and SiO_2_@Au substrates. The characteristic Raman peaks of MB are recorded at 482 cm^−^^1^, 550 cm^−^^1^, 783 cm^−^^1^, and 1082 cm^−^^1^ [44]. The intensities of these peaks are significantly enhanced after coating AuNPs onto SiO_2_ under optimal conditions of synthesis process compared to SiO_2_ particles. 

## 4. Discussion

Our results demonstrate that optimizing parameters for the fabrication SiO_2_@Au nanoparticles is critical to enhance the Raman signal. The results obtained under various synthesis conditions are discussed in detail below. 

As shown in Figure 1, the SPR peak position of SiO_2_@Au is red-shifted with a temperature change from 40 to 100 °C. According to *Link* et al. [45], the SPR peak is red-shifted when the particle diameter is increased. The temperature factor strongly affects the size and shape of synthesized AuNPs and SiO_2_@Au nanoparticles. Thus, a suitably high temperature should be ensured during the synthesis process to obtain uniform SiO_2_@Au nanoparticles and enhance the SERS signal.

For temperatures below 80 °C, the maximum extinction wavelength exhibits red shifts from 520 to 525 nm, and the color of SiO_2_@Au solution changes from pale pink to light red, as shown in Figure 1. These SPR peaks correspond to AuNPs with sized in the range of 10 nm to 40 nm. However, the intensities of the SPR peaks, which depend on the number of AuNPs deposited on the SiO_2_ surface, are low because the reduction rate of the Au^3+^ ion in the HAuCl_4_ solution into the Au atom is slow, and size and number of formed AuNPs are small at these temperatures. The growth of AuNPs and the development of the gold shell on the SiO_2_ surface take place simultaneously; a the uniform gold shell is not completely formed at these temperatures. This affects the enhancement efficiency of the Raman scattering signal.

Next, we investigated the synthesis process at higher temperatures in the range of 80 °C to 100 °C. At these temperatures, the reaction process is accelerated, and the Au atoms move quickly, increasing the probability of coalescence of AuNPs. As a result, the number and size of AuNPs rapidly increases. This is also proven by the color change of the samples from red to dark purple. The amplitudes of SPR peaks are enhanced significantly at wavelengths of 525 nm (80 °C), 530 nm (90 °C), and 533 nm (100 °C), corresponding to a size range of 40 to 50 nm. The width of the SPR band is narrow at these temperatures, which is the result of the uniform size and density of AuNPs covering the SiO_2_ surface. Additionally, the reason for the high SPR peaks is that the citrate surface stabilizer around the gold particles is efficient a suitable temperature, avoiding flocculation and forming reasonably sized AuNPs. With suitable temperatures, the surface density of AuNPs deposited on SiO_2_ spheres is sufficiently dense, which leads to the superposition of electric fields from the close AuNPs and the formation of so-called “hot spots”. This facilitates the enhancement of the Raman signal of synthesized SiO_2_@Au nanoparticles.

However, the temperature is too high, and the reaction speed is too fast, resulting in flocculation [11]. Therefore the properties of the citrate surface stabilizer are changed, the citrate-anion layer capping the gold particles is not efficient at excessively high temperatures, forming gold clusters, and irregularly large sizes and excessively dense surface densities occur. This causes a reduced efficiency of the enhancement of the Raman signal on the SiO_2_@Au [45,46]. Based on the above analyses, 80 °C was selected as the optimal temperature, mainly due to the high amplitude of the SPR peak at the wavelength of 525 nm, the narrow width of the SPR band, and the reasonable AuNPs size of 40 nm at this temperature, contributing to the enhancement of the Raman scattering signal. The optimal temperature of 80 °C was used in synthesis preparation of SiO_2_@Au to investigate the other factors.

The time for SiO_2_@Au synthesis is one of the significant conditions necessary to enhance the intensity of the SERS signal. Figure 2 presents the extinction spectrum of SiO_2_@Au versus time. For a synthesis time of 3 h to 4 h, there is a red-shift of SPR peaks in the extinction spectrum. However, the amplitudes of SPR peaks are low, and the characteristic color of the SiO_2_@Au solution changes to light red, as fewer AuNPs are produced for a short time. For longer reaction times, from 5 h to 6 h, the SPR peaks significantly enhance within the range of 524 to 525 nm, and the color of the samples turns dark red, indicating that with a longer reaction time, more AuNPs are produced, leading to an increase in the SPR peak intensity, as shown in Figure 2c. Moreover, AuNPs have more time to attach to the SiO_2_ spheres with a longer reaction time, which enables SiO_2_@Au to be synthesized more efficiently. Nevertheless, when the reaction time is too long, the gold can be oxidized, decreasing the surface free energy of AuNPs. Thus, the Raman signal of the SiO_2_@Au nanoparticles decreases. In this study, the sizes of AuNPs change in the range of approximately 30–40 nm, and the position of SPR peaks is within the range of 524 to 525 nm for a synthesis time of 3 to 7 h. Because the intensity is unchanged after 6 h, we selected 6 h as the optimal time to reduce the processing time.

Similarly, the ratio of HAuCl_4_ to sodium citrate plays a critical role in determining the size, number, and size distribution of AuNPs on SiO_2_ spheres. The SPR peaks of the obtained SiO_2_@Au nanoparticles occur within the range of 523 nm to 525 nm with a ratio of precursors between 1:104 and 1:21, as shown in Figure 3 and AuNPs in the size range of 20 nm to 40 nm [45,46]. The samples’ color becomes dark purple, and the SPR wavelength is shifted to 536 nm when the ratio of HAuCl_4_ to sodium citrate is 1:15. This indicates that AuNPs with a molar ratio of precursors of 1:15 is larger than particles with other ratios of precursors [45,46]. Moreover, the width of SPR bands at this ratio is wider than the at other ratios as a result of the non-uniform sizes and surface densities of AuNPs. In this study, the optimal ratio of HAuCl_4_ to sodium citrate was selected as 1:21, which is consistent with the size of AuNPs of 40 nm and an SPR peak position of 525 nm.

The sodium citrate concentration influences the formation of AuNPs on SiO_2_ spheres. The reaction of HAuCl_4_ and sodium citrate is presented in Equation (1). According to this equation, the reaction rate depends on both the concentration of HAuCl_4_ and Na_3_C_6_H_5_O_7_. HereinNa_3_C_6_H_5_O_7_ plays not only the role of a reducing agent but also that of a stabilizer [47]. According to Le Chatelier’s principle, the number of AuNPs will increase when the concentration of HAuCl_4_ is increased. However, flocculation also occurs if the amount of stabilizer is not sufficient for the synthesis process. Therefore, the concentration of experimental sodium citrate is higher than the concentration of reacted sodium citrate, i.e., about 50% [47].

2HAuCl_4_ + 3Na_3_C_6_H_5_O_7_ → 2Au + 3Na_2_C_5_H_4_O_5_ + 3CO_2_ + 3NaCl + 5HCl (1)

The SPR peak positions are red-shifted, and their amplitudes are enhanced at high sodium citrate concentrations, accompanied by a noticeable color change in samples, as shown in Figure 4. For the low sodium citrate concentrations of 0.21, 0.42, and 0.84 mM, the intensities of SPR peaks are low, and the color of synthesized solutions changes to pink. This shows that the amounts and size of formed AuNPs are small. The size and number of AuNPs increase with concentrations from 1.5 to 4.2 mM, resulting in AuNPs being deposited on the SiO_2_ surface faster [48]. SPR peaks occur within the range of 523–525 nm, and the samples’ color becomes dark red, consistent AuNPs sizes in the range of 20 to 40 nm [45,46]. A higher sodium citrate concentration facilitates the reduction and subsequent capping of surface-stabilizing agents on AuNPs. Hence, the shape and size of the synthesized SiO_2_@Au nanoparticles are more well-defined and uniform. For the SiO_2_@Au synthesis, a concentration of 4.2 mM is selected because it results in a high SPR peak intensity at the wavelength of 525 nm, a narrow width of the SPR band, and AuNPs with a size of approximately 40nm. Significant enhancement of the Raman scattering signal can take place at this optimal concentration. 

According to the DLS results and SEM images, the size of SiO_2_ particles is smaller than that of SiO_2_@AuNPs. However, the size determined using the DLS method is larger than that determined with SEM images because the diameter in this method is a hydrated diameter, which differs from SEM images. 

A comparison the UV-Vis spectra of SiO_2_, SiO_2_@PEI, AuNPs, and SiO_2_@Au synthesized under optimal conditions shows that a blue shift occurs when AuNPs are coated on the surface of SiO_2_ spheres, confirming that surface plasmon resonance occurs on SiO_2_@Au nanomaterial. AuNP coating on SiO_2_ material can improve and enhance SPR intensity. This finding is consistent with Ref. [49]. In particular, the enhancement of the Raman signal to one typical for SiO_2_ coating gold nanoparticles, compared to SiO_2_ particles, demonstrates the potential applications of SiO_2_@Au, such as environment and food analysis and biomedicine. 

## 5. Conclusions

In summary, SiO_2_@Au was successfully synthesized by a chemical reduction reaction method under optimal conditions of factors affecting the synthesis process, including a ratio of HAuCl_4_ to sodium citrate of 1:21 and an initial concentration of sodium citrate of 4.2 mM at 80 °C for 6h. The obtained SiO_2_@Au particles presented a core shell, in which SiO_2_ particles were coated by AuNPs (approximately 40 nm in size). The initial result shows that an SiO_2_@Au substrate can significantly enhance the Raman intensities compared to an SiO_2_ substrate that is not coated with gold nanoparticles. An analysis of SiO_2_@Au nanoparticles by Raman spectroscopy demonstrated that the enhancement of the surface Raman signal achieved maximum values at 482 cm^−1^, 550 cm^−1^, 783 cm^−1^, and 1082 cm^−1^. This method can be considered a prospective solution for the development of Raman spectroscopy for efficient applications in sensors and catalysis.

## Figures and Tables

**Table 1:** A review of the methods and related conditions for the fabrication of SiO_2_@Au.

Method	Reducing Agents	Functionalization Agents	Reducing Reaction Temperature	Reducing Reaction Time	Ratio of Precures	Concentration of Reducing Agent	Ref
Electroless plating	NH_2_O. HCl	APTMS	-	14 min	-	0.4 mM	[26]
Self-assembly	CH_2_O	APTMS	-	-	-	0.36 mM	[27]
Layer by layer	NaBH_4_	MPTMS	-	6 h	-	-	[28]
Seed-mediated growth	CH_2_O	APTES	-	10 min	-	-	[29]
Ultrasound-assisted Stober method	NaBH_4_	-	20 °C	2 h	-	10 mM	[30]
Direct growth of gold shell	Ascorbic acid	-	-	-	-	3 mM	[32]
Direct growth of gold shell	2-methylaminoethanol	-	80 °C.	30 min	-	-	[33]
Seed-mediated growth	NH_2_OH·HCl	APTES	-	-	8:1 (Na_3_Cit/HAuCl_4_)	40 mM	[37]
Seed-mediated growth	NH_2_OH·HCl + NaBH4	APES	-	-	-	-	[39]
Seed-mediated growth	Na_3_(C_6_H_5_O_7_) + NaBH_4_	APS	-	60 min	0.002 M:0.01 M (HAuCl_4_/ NaBH_4_)	0.01 M	[40]
Seed-mediated growth with grafting, priming technique	NaBH_4_	APTMS	-	12 h	-	5.3 mM	[41]
Ultrasound-assisted seed growth	NH_2_O. HCl	PEI	-	5 min	-	-	[42]
Seed-mediated growth for synthesis of freckled SiO_2_@Au NCs	NaBH_4_	MPTMS	-	10 min	8:1 (K-gold/SiO_2_)	5.3 mM	[43]

## Data Availability

Not applicable.
